# No Effect of Acute or Chronic New Zealand Blackcurrant Extract on Cycling Performance or Physiological Responses in Trained Cyclists

**DOI:** 10.1002/ejsc.12267

**Published:** 2025-02-05

**Authors:** Lillian C. Morton, Carl D. Paton, Ryan Aberkane, Andrea J. Braakhuis

**Affiliations:** ^1^ Department of Nutrition and Dietetics Faculty of Medical & Health Science The University of Auckland Auckland New Zealand; ^2^ School of Health and Sport Science The Eastern Institute of Technology Napier New Zealand; ^3^ Human Biology and Nutrition Department at AgroParisTech The Paris Institute of Technology for Life Food and Environmental Sciences Paris France

## Abstract

Previous research examining the ergogenic benefits of blackcurrant supplementation (BC) on exercise performance is contradictory. The BC supplementation period in many studies has typically been chronic (> 6‐days), with a final dose taken hours before testing. Whether any observed performance benefits are from the acute dose or chronic supplementation is unclear. This study aimed to examine the effects on cycling performance of a single‐acute dose and 7‐day supplementation with BC extract. This study was a placebo‐controlled, double‐blind, randomised, cross‐over trial. Sixteen cyclists (mean ± SD: age 37 ± 11 years; height 175.8 ± 7.6 cm; body mass 73.2 ± 12.5 kg; V̇O_2peak_ 3.8 ± 0.7 L·min^−1^) completed a total of six experimental sessions (2 × 3 treatment blocks). Each treatment block consisted of a no‐supplement baseline trial, a single dose (acute) and a 7‐day (chronic) supplementation trial. During each trial, subjects completed a maximal incremental test and 4 km time trial, separated by 15 min of recovery. Respiratory data, heart rate, muscle oxygenation and performance power were measured continuously in each trial, and differences between treatments were determined using RM‐ANOVA and effect size analysis. There was no significant difference (*p* > 0.05) in cycling performance between experimental and placebo treatments following acute or chronic supplementation periods. There were no significant effects on measured physiological and metabolic parameters, and any observed differences in performance or physiology were trivial to small. Blackcurrant supplementation delivered either acutely or over a period of 7 days had no significant effect on cycling performance or physiology.

**Trial Registration:**

Australia New Zealand Clinical Trial Registry: ACTRN12622001277730

**Summary**

- Dietary antioxidant supplementation use by athletes has increased as a strategy to mitigate training‐induced oxidative stress and inflammation. However, previous research examining the benefits to exercise performance from blackcurrant supplementation is equivocal.
- The supplementation period in previous studies has typically been chronic (> 6‐days), with a final dose often taken hours before testing. It is therefore unclear if any observed performance benefits are from the acute dose or the chronic supplementation.
- No effects in cycling performance, physiology or metabolic measures were observed between BC extract and placebo conditions, or between acute and 7‐day BC intake.

## 1 Introduction

The increased oxidative metabolism that occurs during exercise results in unavoidable increased reactive oxygen species (ROS) and nitrogen species (RONS) (Hurst et al. 2019). Dietary antioxidant supplementation use by athletes has increased as a strategy to mitigate training‐induced oxidative stress and inflammation (Braakhuis, Somerville, and Hurst 2020). A key dietary antioxidant source is Blackcurrants (*Ribes nigrum*), which are naturally high in polyphenols, particularly a flavonoid class called anthocyanins (Watson et al. 2015). Anthocyanins exert antioxidant (Kähkönen and Heinonen 2003), and anti‐inflammatory activity (Tabart et al. 2012), and have been shown to improve insulin sensitivity in healthy individuals (Nolan et al. 2021). Furthermore, peripheral blood flow is improved following blackcurrant intake (Matsumoto et al. 2005), which is attributed to anthocyanins increasing vasodilation. Anthocyanins have also been shown to upregulate endothelial nitric oxide synthase (eNOS) (Xu, Ikeda, and Yamori 2004), and increase fat oxidation, which would have a glycogen sparing effect (Strauss, Willems, and Sheppard 2018). These benefits have been found with various anthocyanin‐rich berries and fruit, such as Montmorency cherry and blueberry, and not blackcurrant exclusively (Copetti et al. 2022). The physiological effects exerted by blackcurrant anthocyanins should theoretically therefore offer benefits to exercise performance beyond their effects on oxidative stress.

Anthocyanins are rapidly broken down in the lumen of the gastrointestinal tract but are retained after intestinal absorption (Kalt et al. 2017), and a larger single daily dose has been shown to result in greater anthocyanin absorption than smaller more frequent dosing (Kalt et al. 2017). Blood plasma levels of anthocyanins have been shown to peak 60 min (Röhrig et al. 2019) to 120 min (De Ferrars et al. 2014; Hurst et al. 2019) following ingestion, with physiological effects observed in a dose‐dependent manner (Hurst et al. 2019; Kalt et al. 2017).

A meta‐analysis on the effect of blackcurrant supplementation on exercise performance has reported a significant improvement in performance following 7‐days of supplementation with intakes between 105 and 210 mg^−1^·day of blackcurrant anthocyanins (Braakhuis, Somerville, and Hurst 2020). The supplementation period in previous studies has typically been chronic (> 6‐days), with a final dose often taken hours before testing. Therefore there is no clear differentiation between acute and chronic doses and it is unclear if any observed performance benefits are from the acute dose or the chronic daily supplementation.

Previous research has shown improvements to exercise performance following blackcurrant extract supplementation. A 2.4% improvement in 16.1‐km cycle time trial performance has been reported in trained cyclists following 7‐days of blackcurrant extract supplementation (105 mg^−1^·day anthocyanins) (Cook et al. 2015). Similarly, Murphy, Cook, and Willems (2017) reported an improvement of ∼1% in the total time to complete two 4‐km cycling time trials with blackcurrant extract supplementation (105 mg^−1^·day anthocyanins), and Perkins et al. (2015) found a 10.6% increase in total distance covered in repeated sprint efforts in runners following blackcurrant extract supplementation. However, blackcurrant extract supplementation (105 mg^−1^·day) did not change critical speed or time to exhaustion during running performance in a study by Pastellidou et al. (2021), nor 16.1 km cycling time trial performance under hypoxic conditions following supplementation of 210 mg^−1^·day anthocyanins (Willems et al. 2019). A dose of either 105 mg^‐1^·day or 210 mg^−1^·day also had no benefits on 16.1 km cycling time trial performance after supplementation (Montanari et al. 2020).

Investigations examining the effects of acute blackcurrant extract supplementation on exercise performance are limited. No performance benefits were found after an acute dose of blackcurrant extract on 16.1 km cycling time trial performance (Montanari et al. 2020; Montanari, Blacker, and Willems 2023), or repeated 8 × 5 min high‐intensity cycling efforts following a high anthocyanin blackcurrant drink (Paton et al. 2022), However, 5‐km running performance was improved following acute supplementation of 900 mg BC extract in a study by Moss et al. (2023).

Supplement and nutrition strategies for athletes are typically prescribed as relative amounts (either mg/kg or g/kg), and many treatment studies take into account mass. Research has shown a dose‐dependent increase in blackcurrant anthocyanin bioavailability from blackcurrant extract consumption, where 3.2 mg/kg body mass was shown to preserve neutrophil function compared to 0.8 mg/kg body mass (Hurst et al. 2019). Given the inconsistencies in previous research findings, and the lack of studies directly comparing acute and chronic blackcurrant supplementation simultaneously, the objective of this study was to determine the effects of a single‐acute dose and 7‐day supplementation with BC extract, prescribed at a dose of 4.3 mg anthocyanins/kg body mass, on cycling time trial performance in trained cyclists. We hypothesise that there will be no effect on exercise performance and physiology from blackcurrant supplementation.

## 2 Methods

### 2.1 Experimental Design

**FIGURE 1:**
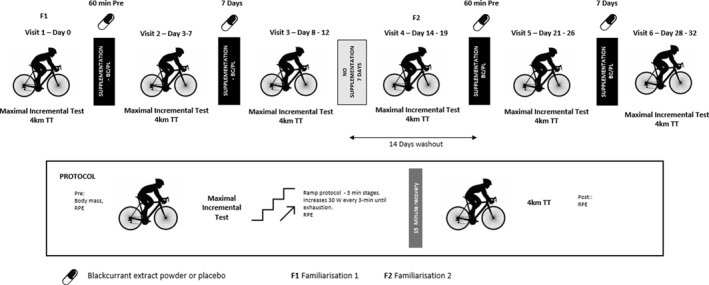
Study schematic outlining the study design and exercise protocol.

The study was a randomised, placebo‐controlled, double‐blind, cross‐over trial. Trial order was prescribed using a balanced Latin square design by an independent assessor who was not involved in the supplement delivery or study data collection. There were two separate experimental treatments consisting of acute and chronic blackcurrant extract supplementation, and acute and chronic placebo supplementation. Prior to each treatment arm, participants completed two baseline trials with no supplement to assess possible training effects. The study design is depicted in Figure 1.

### 2.2 Participants

Trained male and female cyclists were recruited from local cycling clubs within the region. Participants were required to have a training history of > 2 years, no chronic health conditions, or respiratory conditions (i.e., asthma or exercise‐induced asthma or breathlessness), no acute respiratory illness within the last month, no medications that affect respiratory, cardiovascular, or immune function, and be over 16 years of age.

All subjects gave written informed consent after the experimental procedures, associated risks, and potential benefits were explained before the commencement of study participation. The study was approved by the Northern Health and Disability Ethics Committee (2022 EXP 13363), registered with the Australia New Zealand Clinical Trial Registry (ACTRN12622001277730) and conformed to the 2013 Declaration of Helsinki.

An a priori sample size of 16 participants was estimated using power of 0.80, and alpha of 0.05, and the statistical freeware G*Power (version 3.1; Faul et al. 2009), using an effect size (*d* = 0.36) for sample size estimation from previously observed mean ± SD differences between NZBC extract and PL in cycling performance (Cook et al. 2015).

### 2.3 Experimental Sessions

Participants reported to the laboratory on six separate occasions. Experimental sessions were separated by 7 days and conducted at the same time of day for each participant to control for diurnal variation. Participants were instructed to avoid strenuous physical activity and replicate any light training performed in the 24 h before each testing session. Participants were also required to self‐report their dietary intake for the 24 h preceding the first experimental trial and replicate this as closely as possible for each subsequent trial. Participants were required to abstain from any caffeine‐containing products in the 12 h before a test. Participants refrained from eating and only drank water in the 2 h before each testing session commenced. Participants were weighed when they arrived at the laboratory and fitted with sundry measurement equipment.

The exercise protocol consisted of a maximal incremental test, followed by 15 min of rest. Thereafter, participants completed a 4 km time trial (TT) as fast as possible. Cyclists completed testing on a calibrated Velotron Dynafit Pro cycle ergometer (RacerMate Inc, WA, USA) using the company's associated software package. The cycle ergometer was adjusted to a position that resembled the set‐up of the participant's own racing bicycle, and the selected dimensions were recorded and replicated for subsequent tests. The incremental test started at 100 W for male cyclists and 70 W for females and increased by 30 W every 3 min until cyclists reached volitional exhaustion. Incremental exercise protocols comprising 3‐min stages provide the most reliable and valid measures of endurance performance, and are therefore recommended during pre‐experimental testing (Bentley, Newell, and Bishop 2007). Participants were required to remain seated throughout the test and maintain a cadence of 80–100 rpm. Indicators of achieving V̇O_2peak_ were volitional fatigue, a plateau in V̇O_2_, a drop in cadence below 80 rpm, and/or a RER ≥ 1.15. Ratings of perceived exertion (Borg RPE 6–20 scale) (Borg and Kaijser 2006), were obtained in the last 15 s of each stage of the ramp test. On completion of the maximal incremental test, participants had 15 min of recovery. In the final 5 min of the recovery period, cyclists returned to the cycle ergometer and cycled lightly (< 70 W). At the end of the 15‐min recovery period, participants immediately commenced a self‐paced maximal effort 4 km TT. All cycling data (time, distance, power, speed) was captured by the ergometer's associated software package (RacerMate Inc, WA, USA). Participants could only view their gearing and accumulated distance covered but were blinded to any other information to reduce potential pacing effects. During the experimental trials, participants were cooled with standing floor fans and permitted to drink water ad libitum in the rest period and post the 4 km TT. Participants reported their RPE immediately after completing the 4 km TT and then dismounted the ergometer.

During all testing, respiratory gases and heart rate were measured continuously with a calibrated metabolic system (Metamax, Cortex, Leipzig, Germany) using breath‐by‐breath recording mode. Peak power output (PPO) achieved was defined as the mean power during the final 30‐s of the test; V̇O_2peak_ was defined as the highest V̇O_2_ measured over any 30‐s continuous period during the test. All experimental trials were conducted in an environmentally controlled laboratory (temperature 19°C ± 1°C: relative humidity 36% ± 6%). The first visit to the lab served as an initial familiarisation trial with no supplementation of either PL or BC. Subjects were then given their supplement allocated by an individual independent of the study. Neither the researcher nor the subjects were aware of the intervention allocation. Participants were required to consume the ‘acute’ dose of their supplement 60 min before their arrival time at their next lab visit 7 days later. The total time from supplement consumption to the start of the experimental trial was approximately 90 min. Following 7‐days of supplementation participants returned to the lab and completed the experimental protocol. This acted as the ‘chronic’ trial. Seven days later, participants returned to the lab to complete a second familiarisation trial with no supplementation of either PL or BC. As the study period was 6 weeks in duration, the second familiarisation trial at this stage served as an opportunity to determine if changes to V̇O_2peak_ and therefore fitness levels had changed over the previous 3 weeks. This resulted in a 14‐day washout period between the chronic and acute supplementation trials. A summary of the study protocol can be found in Figure 1.

### 2.4 Supplementation

Subjects consumed either blackcurrant extract (BC) or PL. Both the BC and the PL were encapsulated into berry‐flavoured, purple gelatine capsules (The Capsule Guy, Adelaide, Australia) to ensure the colour and odour of the blackcurrant were undetectable to participants. Supplements were given to the researcher on the day of testing by a person independent of the study to be given to participants on the day of testing closest to the acute trial. The BC was prescribed as a dose of 4.3 mg^−1^.kg body mass anthocyanins daily (mean dose ± SD: 315 ± 54 mg), and capsules were individually prepared for each participant. Supplement and nutrition strategies for athletes are typically prescribed as relative amounts (either mg/kg or g/kg bodyweight), and many treatment studies take into account body mass, hence our decision to prescribe anthocyanins as a relative rather than absolute dose. Only one study to our knowledge has used this strategy with blackcurrant extract (Hurst et al. 2019) hence our decision to prescribe anthocyanins as a relative rather than absolute dose. Based on findings in previous research (Hurst et al. 2019; Kalt et al. 2017), we chose to utilise a 4.6 mg/kg dosing strategy in the present study. The placebo was dextrose (< 1 g), and the number of capsules matched those required for the anthocyanin dose. The researcher remained blinded to the interventions throughout data collection. Participants were verbally asked at each visit which supplement they believed they had taken.

### 2.5 Muscle Oxygenation

Skeletal muscle oxygenation (SmO_2_) of the vastus lateralis of the right leg was continuously measured using a near‐infrared spectroscopy (NIRS) sensor (Moxy monitor, Fortiori Design LLC, Minnesota, USA). The sensor was firmly attached at half the length of the vastus lateralis (greater trochanter to lateral knee joint space) in a black light‐blocking case to reduce light interference. The transmitted light was recorded at 0.5 Hz and received into proprietary software (PerfPro Software v 5.82.06, Hartware Technologies, Rockman, MI). Data was interpolated into 1‐min intervals and averaged over 3 min for the maximal incremental test and 1 min for the 4 km TT.

### 2.6 Determination of Ventilatory Threshold

Visual estimates of VT_1_ and VT_2_ were determined independently by two researchers (LM and CP) by plotting ventilation (V̇E) against exercise time (Neder and Stein 2006). Both were blinded to subject identification and the VTs determined by the other researcher. The 15 s rolling data of ventilation (V̇E) from the incremental test were plotted graphically for inspection. The reviewers considered a plot of the V‐slope of V̇E and time for determining VT_1_ and VT_2_. The corresponding power output at which the first clear breakpoint (beginning of increase) of V̇E was considered VT_1_, and the second breakpoint VT_2_. The ventilatory thresholds were expressed as absolute power output and as a percentage of PPO (VT_PPO_).

### 2.7 Data Analysis Procedures and Statistical Analysis

Breath‐by‐breath data for V̇O_2_, V̇CO_2_, RER, V̇E, BF and *V*_*T*_ from each test were examined for errant breaths and outliers (> 3 SDs from the mean) removed. The breath‐by‐breath data was averaged into 1‐min intervals for each minute of the incremental test, and 2‐min steady state data from each stage used for analysis of metabolic variables. Statistical analyses were conducted using GraphPad Prism version 9.3.0 for Windows (GraphPad Software, San Diego, California USA). The normality of data residuals was confirmed using the Shapiro‐Wilk test prior to all analyses, and Greenhouse‐Geiser adjustments were applied for violations (*p* < 0.05). Differences between treatments were determined using a one‐way repeated measures analysis of variance (ANOVA), or via a two‐way repeated measures ANOVA (treatment × time). Where significance was detected, Tukey's multiple comparisons test post‐hoc analysis was used to compare differences between treatments in one‐way repeated measure ANOVAs. Statistical significance was defined as *p* ≤ 0.05. The magnitudes of the standardised effects for the test measures were also determined using the Cohen effect size (*d*). Thresholds of 0.2, 0.5, and 0.8 for small, moderate, and large effects, respectively, were used in accordance with the recommendations of Cohen (1988). ES values < 0.2 were deemed trivial differences. Simple group statistics are shown as means ± SD, unless otherwise stated. Percentage (%) differences between treatments are reported as mean ± 95% confidence intervals (CI).

## 3 Results

### 3.1 Participants

Sixteen trained cyclists, 10 males (mean ± SD: age, 35.6 ± 12.0 years; height, 177.3 ± 8.6 cm; body mass, 74.0 ± 12.1 kg; V̇O_2peak_ 4.14 ± 0.62 L·min^−1^, V̇O_2peak_ 56.55 ± 6.62 mL·kg^−1^, PPO 374 ± 47 W, rPPO 5.17 W/kg) and six females (mean ± SD: age, 38.3 ± 8.4 years; height, 173.3 ± 4.6 cm; body mass, 71.86 ± 13.1 kg; V̇O_2peak_ 3.30 ± 0.45 L·min^−1^, V̇O_2peak_ 47.11 ± 9.60 mL·kg^−1^, PPO 373 ± 46 W, rPPO 4.12 W/kg) volunteered to participate in this study. All cyclists were competitive at club level and trained a minimum of 5 days per week. Based on the average VO_2peak_ (L·min^−1^) and peak power output (PPO W), male cyclists were classified as performance level 2 (PL2) (De Pauw et al. 2013), and female cyclists performance level 4 (PL4) (Decroix et al. 2016).

### 3.2 Four Kilometres Cycling TT Performance

**FIGURE 2:**
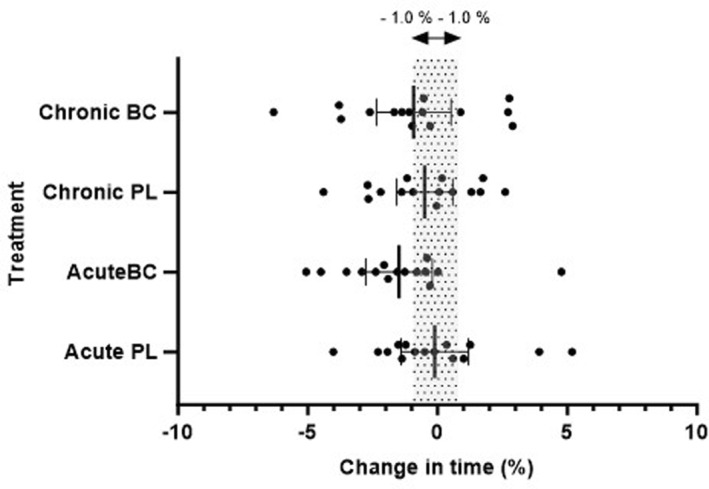
Mean (± 95% confidence limit) change in 4 km TT performance time as percent (%) for different treatment conditions. Shaded area represents ± 1%. Closed circles (•) are individual participant responses.

**FIGURE 3:**
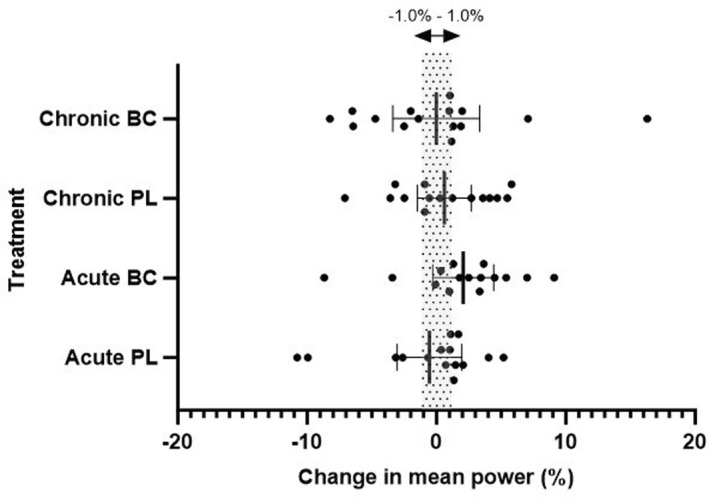
Mean (± 95% confidence limit) change in mean power output in the 4 km TT as percent (%) for different treatment conditions. Shaded area represents ± 1.0%. Closed circles (•) are individual participant responses.

Due to a technical issue with the cycle ergometer, one subject (male) was excluded from analysis of the 4 km TT analysis. The averaged coefficient of variation (CV) for the two baseline trials was 1.6% for male cyclists and 2.8% for female cyclists. The combined CV for both genders was 2.6%. No differences in 4 km TT performance time were observed between BC and PL after either acute (*p* = 0.44), or chronic supplementation (*p* = 0.96). Average power output in the 4 km TT was not statistically significant across trials or between treatment arms (*p* > 0.05), and effects were trivial to small (Table 1). The % change in 4 km TT performance time and average power output relative to the baseline trials can be found in Figure 2, and average power output in Figure 3. There were no statistically significant differences in the average power output in the 4 km TT relative to the PPO achieved in the preceding incremental test, however a small effect was seen with acute BC supplementation (*d* = 0.35) (Table 2).

**TABLE 1:** Maximum power output (W) achieved in the maximal incremental test, and ventilatory thresholds following acute and 7‐day supplementation with PL and BC.

	Acute PL	Acute BC	Chronic PL	Chronic BC	Acute PL versus acute BC	Chronic PL versus chronic BC
V̇O_2peak_ (L·min^−1^)	3.66 ± 0.77	3.70 ± 0.76	3.73 ± 0.75	3.71 ± 0.73	−0.04 [−0.26, 0.17] *d* = 0.05	0.03 [−0.18, 0.24] *d* = 0.03
HR (bpm)	175 ± 12	175 ± 11	173 ± 11	174 ± 11	−0.07 [−6.69, 5.55] *d* = 0.00	−1.29 [−6.90, 4.33] *d* = 0.09
PPO (W)	345 ± 64	344 ± 64	346 ± 60	347 ± 60	0.73 [−7.84, 9.30], *d* = 0.02	−1.20 [−9.98, 7.58], *d* = 0.02
VT1 (W)	190 ± 54	187 ± 54	188 ± 54	191 ± 51	2.64 [−6.87, 12.17] *d* = 0.05	−3.24 [−12.77, 6.27] *d* = 0.06
VT2 (W)	261 ± 58	260 ± 57	258 ± 58	260 ± 56	1.36 [−8.64, 11.36] *d* = 0.02	−2.78 [−12.78, 7.22] *d* = 0.05
VT1% PPO	55 ± 8	54 ± 7	54 ± 9	55 ± 8	0.55 [−2.85, 3.96] *d* = 0.07	−0.66 [−4.06, 2.75] *d* = 0.08
VT2% PPO	75 ± 9	75 ± 6	74 ± 7	75 ± 7	0.10 [−3.68, 3.88] *d* = 0.02	−0.32 [−4.10 to 3.46] *d* = 0.04

*Note:* Data reported as mean ± SD from 15 subjects. Comparisons are reported as Mean difference and 95 CI of the difference between treatment and placebo. % PPO is the percentage of the mean power output in the incremental test.

No differences were observed in heart rate, V̇O_2_ (L·min^−1^), respiratory exchange ratio (RER), or SmO_2_. A summary of ventilatory and gas exchange dynamics during 4 km TT cycling performance can be seen in Table 2.

**TABLE 2:** Four‐Kilometres TT cycling performance time, average power output, and ventilatory and gas exchange dynamics following acute and 7‐day supplementation with PL and BC.

	Acute PL	Acute BC	Chronic PL	Chronic BC	Acute PL versus acute BC	Chronic PL versus chronic BC
4‐km time (s)	374 ± 38	368 ± 36	373 ± 39	371 ± 37	5.31 [−3.59, 14.22], *d* = 0.14	1.80 [−6.09 to 9.70], *d* = 0.05
Avg power (W)	295 ± 62	303 ± 65	299 ± 67	296 ± 64	−7.83 [−20.78, 5.11], *d* = 0.13	2.91 [−10.03, 15.86], *d* = 0.05
% PPO	85 ± 7	88 ± 5	86 ± 7	85 ± 7	−2.27 [7.14, 2.61], *d* = 0.35	0.95 [−3.06, 4.97], *d* = 0.14
V̇O_2_ (L·min^−1^)	3.30 ± 0.75	3.46 ± 0.72	3.30 ± 0.71	3.35 ± 0.65	−0.17 [−0.37, 0.03], *d* = 0.21	−0.05 [−0.34, 0.03] *d* = 0.07
HR (bpm)	163 ± 13	165 ± 12	163 ± 12	163 ± 12	−1.48 [−5.30, 2.34] *d* = 0.16	−0.48 [−4.30, 3.34], *d* = 0.00
RER	0.98 ± 0.04	0.99 ± 0.05	0.98 ± 0.04	0.97 ± 0.05	−0.01 [−0.04, 0.02], *d* = 0.22	1.1 [−0.02, 0.04], *d* = 0.22
SmO_2_ (%)	35.08 ± 16.32	33.42 ± 19.21	30.93 ± 19.03	33.21 ± 18.76	1.66 [−8.23, 11.55], *d* = 0.11	−2.28 [−14.67, 10.12], *d* = 0.10

*Note:* Data reported as mean ± SD from 15 subjects. Comparisons are reported as Mean difference and 95 CI of the difference between treatment and placebo. % PPO is the percentage of the mean power output in the preceding maximal incremental test.

### 3.3 Incremental V̇O_2_ Responses

There were no significant differences between treatment arms in peak V̇O_2_ (*p* = 0.441) or the maximum power output achieved in the incremental test (*p* = 0.334), nor were there any differences in VT_1_ (*p* = 0.56) or VT_2_ (*p* = 0.91) between treatments.

## 4 Discussion

The aim of this research was to compare the effects of a single dose of BC extract with 7‐days of BC extract consumption on cycling performance with trained cyclists. To our knowledge this study is the first to specifically compare the performance effects of acute versus chronic supplementation in a randomised, double‐blind, placebo‐controlled design. The primary results indicate that there were no significant performance benefits to maximal incremental power or 4‐km TT cycling performance compared to placebo from either acute or 7‐day supplementation of BC extract (mean dose 315 mg anthocyanins). Additionally, there were no significant differences in metabolic or physiological variables during an incremental ramp test or 4 km TT between any BC extract treatment regime compared to placebo.

The absence of ergogenic benefit from acute BC supplementation has been observed in previous research findings. Ross et al. (2023), reported no benefit to 10 km cycling performance time, mean power, or tissue oxygen saturation following an acute dose of 900 mg of BC extract (315 mg anthocyanins) 1 h prior to a 10 km cycling TT. Montanari et al. (2020), using an acute dose of 600 mg (210 mg anthocyanins), found no benefit to 16.1 km cycling TT performance, or differences in physiological and metabolic responses. In a further study by Montanari et al. (2023), no differences were seen in a home‐based (ZWIFT) 16.1 km cycling TT following acute consumption of 900 mg of BC extract (315 mg anthocyanins) 2‐h before the exercise protocol.

Despite a lack of statistically significant benefit to time trial performance in the present study, a small effect (*d* = 0.36) was observed in % PPO during the 4 km TT following acute BC supplementation compared to PL. Responses were variable across participants suggesting large inter‐individual differences. Variability in responses to blackcurrant supplementation have been attributed to individual differences in absorption and metabolism, genetic variations in the expression of functional enzymes, or factors such as gender (Eker et al. 2019). The present study included a combination of male and female participants, however it would have been imprudent to explore gender differences given the small number of female participants (*n* = 6). The literature on anthocyanin bioavailability considering inter or intra‐individual variability is scarce and individual responses to BC extract supplementation in exercise performance have yet to be explored.

Anthocyanins are rapidly broken down in the lumen of the gastrointestinal tract but are well retained after intestinal absorption (Kalt et al. 2017), and a larger single daily dose has been shown to result in greater anthocyanin absorption than smaller more frequent dosing (Kalt et al. 2017). Previous research has shown a dose‐dependent increase in blackcurrant anthocyanin bioavailability from blackcurrant extract consumption (Rodriguez‐Mateos et al. 2013; Hurst et al. 2019), which exert benefits to neutrophil count and other inflammatory markers (Hurst et al. 2019). A limitation to this study is that no plasma anthocyanin measurements were made, or biomarkers that reflect antioxidant capacity. The absence of measures of antioxidant status is evident in all BC supplementation and exercise research, and as such, much of the possible mechanisms that underlie any effects are speculative and based on in vitro and murine models, or research exploring health benefits related to anthocyanin intakes.

One of the mechanisms proposed to enhance exercise performance from BC supplementation is the potential for anthocyanins to enhance blood flow (Cook et al. 2015; Cook et al. 2017; Matsumoto et al. 2001; Copetti et al. 2022). Exercise performance is reliant on the delivery of oxygen and substrate to the working muscle, and the removal of metabolic by‐products such as lactate. Enhanced or improved blood flow would therefore be advantageous to the exercising athlete. Anthocyanins mediate nitric oxide availability, which result in changes in vascular tone and resting haemodynamics (Bell and Gochenaur 2006), and acute blackcurrant ingestion has been shown to maintain blood flow during static (Barnes et al. 2020), and isometric (Cook et al. 2022) conditions. However, these conditions do not reflect the dynamic state of muscle metabolism during exercise. Indeed, Barnes et al. (2020), found that despite acute blackcurrant maintaining blood flow during rest, no improvements in subsequent repeated hand‐grip exercise performance were observed. In the present study there were no differences in SmO_2_ between treatments, indicating no differences to oxygen delivery to working muscles from BC supplementation. Measurement of skeletal muscle oxygenation (SmO_2_) reflects localised muscle capillary content, is negatively correlated with both VO_2_ and HR (Crum et al. 2017), and is considered an acceptable index of metabolic demand within the working muscle.

Supplement and nutrition strategies for athletes are typically prescribed as relative amounts (either mg/kg or g/kg bodyweight), and many nutrition treatment studies take into account mass. However few BC intervention trials have utilised relative dosing strategies in humans. Anthocyanin doses of 310 mg anthocyanin have been shown to positively influence FMD and cardiovascular health (Rodriguez‐Mateos et al. 2013), and a dose of 4.3 mg/kg used in this study resulted in a mean intake of 315 mg anthocyanins. This is in alignment with previous research which has administered 900 mg blackcurrant extract acutely (Ross et al. 2023; Montanari et al. 2023). Participants in the present study consumed their acute dose 60 min before arrival at the laboratory as blood plasma levels of BC anthocyanins have been shown to peak between 1 and 2 h following ingestion (Matsumoto et al. 2001; Hurst et al. 2019). Once sundry equipment had been fitted, and the maximal incremental test completed, the 4 km cycling TT was approximately 2 h post ingestion, which is in alignment with previous research.

A unique aspect of the present study was the inclusion of two baseline trials, one before each supplementation period to determine any potential training effects across the 6‐week trial period. Furthermore, the two baseline trials allowed a reliability estimate (CV%) to be calculated for the experimental tests. Both maximal incremental test performance and cycling TT performance showed good reliability (particularly male cyclists CV < 2%) over a period of 6‐weeks, and no differences between experimental conditions were greater than the observed CV. Fifteen participants were included in the final data analysis, and we aimed to include both male and female subjects. However, the study would have benefitted from a greater sample size, particularly female subjects, to allow gender differences to be explored and improve the statistical power of the study. A meta‐analysis by McNulty et al. (2020), has indicated that the effects of the menstrual cycle on exercise performance is highly individual, and the observed effect on cycling performance is trivial (*d* = < 0.2). In the present study, phases of the menstrual cycle were considered in the study design (with the washout period), and menstrual status was confirmed verbally at each visit.

## 5 Conclusion

This study reports that BC extract supplementation delivered as either an acute dose or consumed daily for 7‐days at a dose of 4.3 mg anthocyanins/kg does not benefit incremental exercise or 4 km cycling TT performance when compared with a placebo control. Furthermore, there were no significant differences in physiology or metabolic measures between treatment and placebo conditions, or between acute and 7‐day BC intake. Therefore we conclude that BC supplementation is unlikely to provide an ergogenic benefit to athletes performing short duration high intensity exercise.

## Author Contributions

Conceptualization: L.C.M. and C.D.P. Methodology: L.C.M. and C.D.P. Investigation: L.C.M., C.D.P. and R.A. Formal analysis: L.C.M. Writing—original draft preparation: L.C.M. Writing–original draft: L.C.M. Writing—review and editing: L.C.M., C.D.P. and A.J.B. Visualization: L.C.M. All authors have read and agreed to the published version of the manuscript.

## Ethics Statement

The study was approved by the Northern Health and Disability Ethics Committee (2022 EXP 13363).

## Conflicts of Interest

The authors declare no conflicts of interest.

## Data Availability

The consent given by the participants does not open for sharing the full data set.
